# Supplementary material for: Mistletoe-Extract Drugs Stimulate Anti-Cancer Vγ9Vδ2 T Cells
Source: Cells. 2020 Jun 26;9(6):1560. doi: 10.3390/cells9061560 (PMC7349316; doi:10.3390/cells9061560)
Supplement: Supplementary file 1 [file cells-09-01560-s001.pdf]

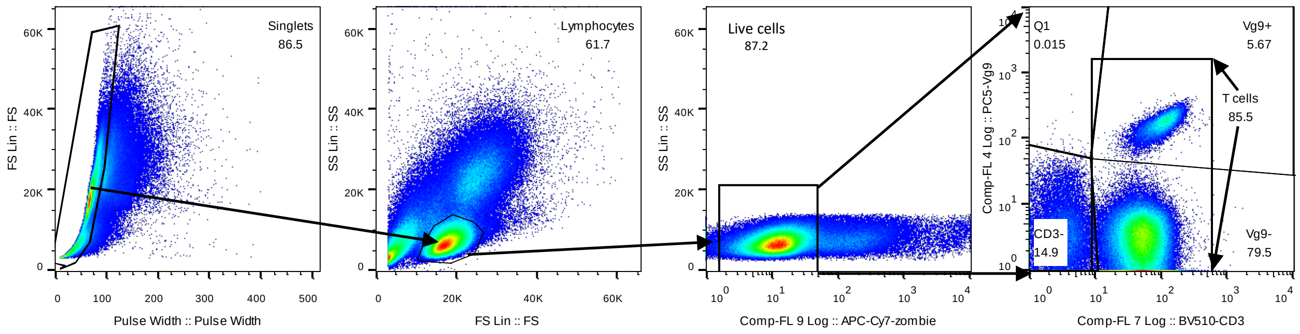

Supplemental Figure 1. Gating strategy for live cells and for the main subsets studied in this article.

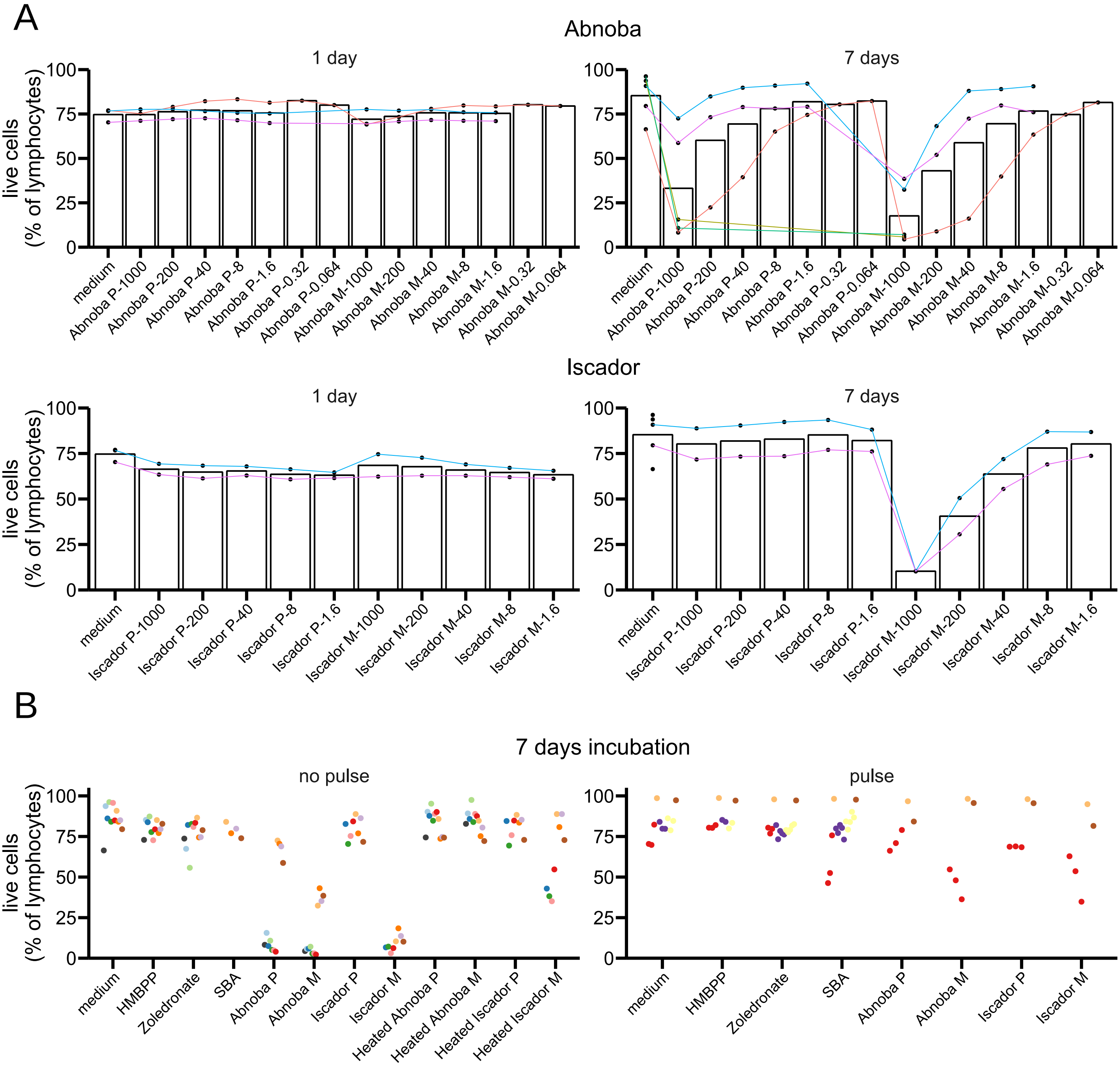

Supplemental Figure 2. In vitro cytotoxicity on PBMCs of each mistletoe-extract drug.
(A) Percentage of lymphocytes that are negative for staining with the Zombie-NIR viability dye. Lines connect the same subjects (n=2-5), bars indicate mean values. Concentrations (in µg/ml) are indicated after the extract names on the X-axis. Incubation times (1 day or 7 days) are indicated.
(B) Zombie-NIR negative percentage of lymphocytes within each stimulation after 7 days incubation. Data are from all the 5 or 7 days tests used in this paper. Lines connect the same subject and each color represent one subject (n=13); each dot represents an independent experiment.

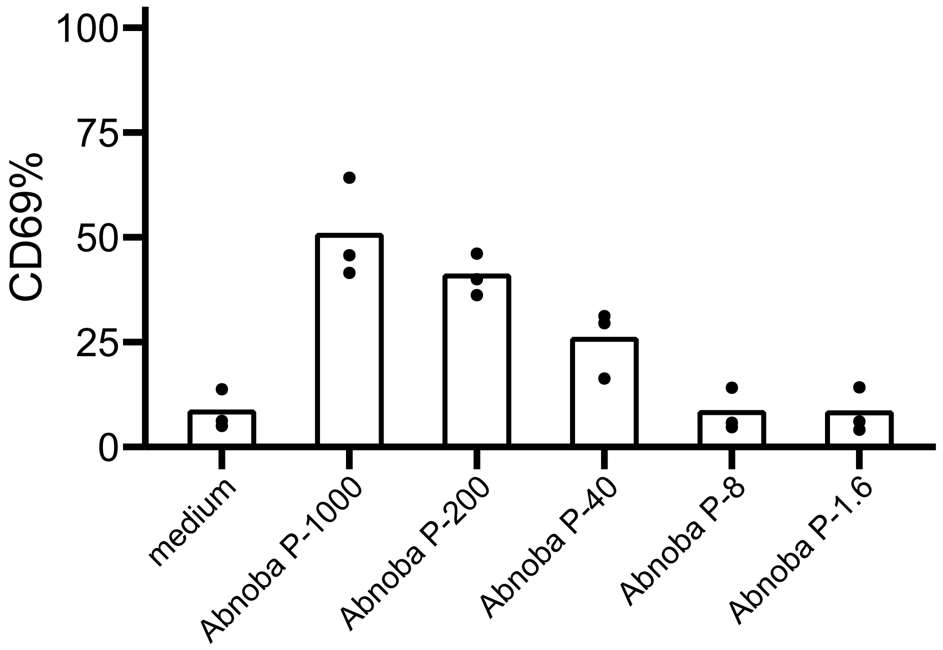

Supplemental Figure 3. CD69 expression on Vγ9+ T cells after 1-day stimulation with different concentrations of AbnobaViscum Pini (Abnoba P).

Bars indicate mean value; concentrations are indicated after the stimulant name on the X-axis. Each dot represent the data of one subject.

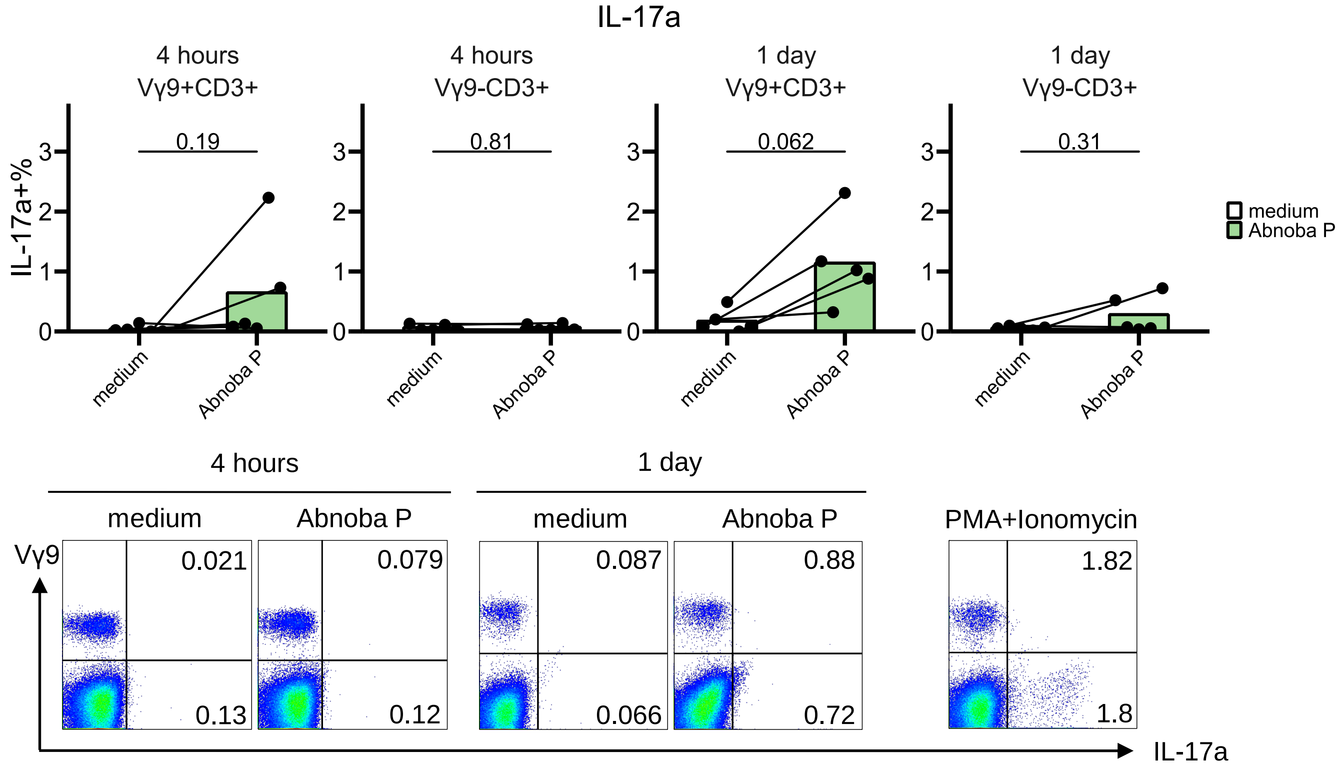

Supplemental Figure 4. Absence of IL-17a induction by AbnobaViscum Pini (Abnoba P) stimulation in Vγ9+ T cells and Vγ9- T cells.

Lines connect the same subject (n=5), bars indicate mean value. Values on the graphs indicate p values (obtained with the Wilcoxon signed-rank test). Bottom panels show representative flow cytometry plots (gate on CD3+ T cells). Upper and lower numbers indicate percentages of IL-17-positive cells in Vγ9+ T cells and Vγ9- T cells respectively. Incubation time (4h or 1 day) is indicated.
